# Exploring relationships between hospital patient safety culture and Consumer Reports safety scores

**DOI:** 10.1186/s12913-017-2078-6

**Published:** 2017-02-16

**Authors:** Scott Alan Smith, Naomi Yount, Joann Sorra

**Affiliations:** Westat, 1600 Research Blvd, Rockville, MD 20850 USA

**Keywords:** Safety culture, Patient safety, Hospital, Survey, Consumer reports, CAHPS

## Abstract

**Background:**

A number of private and public companies calculate and publish proprietary hospital patient safety scores based on publicly available quality measures initially reported by the U.S. federal government. This study examines whether patient safety culture perceptions of U.S. hospital staff in a large national survey are related to publicly reported patient safety ratings of hospitals.

**Methods:**

The Agency for Healthcare Research and Quality Hospital Survey on Patient Safety Culture (Hospital SOPS) assesses provider and staff perceptions of hospital patient safety culture. Consumer Reports (CR), a U.S. based non-profit organization, calculates and shares with its subscribers a Hospital Safety Score calculated annually from patient experience survey data and outcomes data gathered from federal databases. Linking data collected during similar time periods, we analyzed relationships between staff perceptions of patient safety culture composites and the CR Hospital Safety Score and its five components using multiple multivariate linear regressions.

**Results:**

We analyzed data from 164 hospitals, with patient safety culture survey responses from 140,316 providers and staff, with an average of 856 completed surveys per hospital and an average response rate per hospital of 56%. Higher overall Hospital SOPS composite average scores were significantly associated with higher overall CR Hospital Safety Scores (β = 0.24, *p* < 0.05). For 10 of the 12 Hospital SOPS composites, higher patient safety culture scores were associated with higher CR patient experience scores on communication about medications and discharge.

**Conclusion:**

This study found a relationship between hospital staff perceptions of patient safety culture and the Consumer Reports Hospital Safety Score, which is a composite of patient experience and outcomes data from federal databases. As hospital managers allocate resources to improve patient safety culture within their organizations, their efforts may also indirectly improve consumer-focused, publicly reported hospital rating scores like the Consumer Reports Hospital Safety Score.

## Background

Since the Institute of Medicine published *To Err is Human* in 2000 [1], there has been an increase in federal reporting requirements for hospitals. In turn, consumers’ access to a wide range of hospital safety and quality measures, including infection rates, preventable readmission rates, and data on patient experience, has also increased. Multiple private reporting services in the U.S. have aggregated these hospital quality measures into single, easy-to-use hospital scores to help consumers interpret the data when deciding where to seek care—for example, the Consumer Reports (CR) Hospital Safety Score [2], U.S. News and World Report Hospital Rankings [3], and Leapfrog Hospital Safety Score [4]. However, these reporting systems often provide conflicting information [5, 6]. Each rating system uses its own rating methods, focuses on different aspects of quality of care, and measures different areas of performance. It is important for consumers to understand the differences between these systems and for hospitals to understand drivers or ways to improve their scores.

Many hospitals currently assess patient safety culture to identify areas for improvement. Patient safety culture refers to providers’ and staff values, beliefs, and norms about what is important in a healthcare organization, how organization members are expected to behave, what attitudes and actions are appropriate, and what processes and procedures are rewarded and punished with regard to patient safety [7].

CR has more than 3 million paying online subscribers who have access to U.S. Medicare-certified hospital safety and quality measures, including the Hospital Safety Score [8]. The Hospital Safety Score is a composite rating based on publicly available federal data regarding infection and readmissions rates, communication between staff and patients, use of scanning, and mortality rates, and gives consumers a way to compare hospitals on patient safety. Non-subscribers can view the top 10 scoring and bottom 10 scoring hospitals for free online [9]. In addition, local and national news outlets annually cover CR’s release of the Hospital Safety Score, which pressures hospital leaders to publicly respond to their respective ratings and national rankings [10–12].

Given the local and national attention that the CR Hospital Safety Score receives, hospitals may be motivated to improve quality measures that are used to calculate their Safety Score. Focusing on patient safety culture may do just that. Recent studies found relationships between patient safety culture and patient safety indicators and readmission rates, which are used to calculate the CR Hospital Safety Score. Singer et al. found that higher staff perceptions of patient safety culture were related to fewer patient safety adverse events [13]. Similarly, Mardon et al. found that higher patient safety culture scores were associated with fewer adverse events in hospitals [14]. Another study reported lower non-management staff perceptions of safety climate were associated with higher readmission rates for acute myocardial infarction and heart failure, as well as lower rates of mortality [15]. Birkmeyer et al. found that rates of serious complications were significantly lower among hospitals receiving an overall safety rating of “Excellent” from nurses, compared with those receiving a “Very good” or “Acceptable” rating [16].

Patient safety culture has also been linked with patient experience, which is another quality measure used to calculate the CR Hospital Safety Score. Sorra et al. found that hospitals with higher patient safety culture scores tended to have more positive assessments of care from patients on the Consumer Assessment of Healthcare Providers and Systems (CAHPS®) Hospital Survey [17]. Patients’ perceptions of communication with nurses (e.g. explained things so that patients could understand) and responsiveness of hospital staff were most often positively related to staff perceptions of patient safety culture as measured by the Agency for Healthcare Research and Quality (AHRQ) Surveys on Patient Safety Culture (SOPS^TM^) Hospital Survey.

The purpose of this research is to examine the relationship between one of the publicly reported, consumer-oriented hospital patient safety rating systems, the CR Hospital Safety Score, and patient safety culture scores as measured by AHRQ’s Hospital SOPS. We hypothesized that hospitals with higher Hospital SOPS scores would have a higher CR Hospital Safety Score. Furthermore, we hypothesized that hospitals with higher Hospital SOPS scores would be related to the CR Hospital Safety Score components in the following ways: 1) lower hospital acquired infection rates, 2) lower hospital-wide readmission rates, 3) better communication about medications and discharge with patients, 4) more appropriate use of scanning, and 5) lower mortality rates.

## Methods

### Data sources and measures

#### AHRQ Hospital Survey on Patient Safety Culture (Hospital SOPS)

The Hospital SOPS assesses provider and staff perceptions about patient safety issues, medical errors, and event reporting. The development team reviewed research pertaining to safety, patient safety, health care quality, medical errors, error reporting, and organizational climate and culture. In addition, existing safety climate and culture instruments were examined. Then, key dimensions of patient safety culture were identified and survey items were developed. The survey was pilot tested, revised, reviewed by technical experts and then AHRQ released it in 2006. The survey is psychometrically sound at the individual, unit, and hospital levels of analysis [7].

The survey includes 42 items that measure 12 composites of patient safety culture. Each of the 12 patient safety culture composites is listed and defined in Table 1. The items that make up these composites and the overall patient safety rating item can be found on the AHRQ Web site [18]. AHRQ funds a comparative database to enable hospitals to compare their survey results with other hospitals [19]. Hospitals voluntarily submit their data for inclusion in the database.

**Table 1:** AHRQ Hospital Survey on Patient Safety Culture Composites and Definitions

Patient safety culture composite	Definition: The extent to which	Number of survey items
1. Communication openness	Staff will freely speak up if they see something that may negatively affect patient care, and feel free to question those with more authority	3
2. Feedback & communication about error	Staff are informed about errors that happen, given feedback about changes put into place based on event reports, and discuss ways to prevent errors	3
3. Frequency of events reported	Mistakes of the following types are reported: 1) mistakes caught and corrected before affecting the patient, 2) mistakes with no potential to harm the patient, and 3) mistakes that could harm the patient, but do not	3
4. Handoffs & transitions	Important patient care information is transferred across hospital units and during shift changes	4
5. Management support for patient safety	Hospital management provides a work climate that promotes patient safety and shows that patient safety is a top priority	3
6. Nonpunitive response to error	Staff feel that their mistakes are not held against them, and mistakes are not kept in their personnel file	3
7. Organizational learning—Continuous improvement	Mistakes have led to positive changes and changes are evaluated for their effectiveness	3
8. Overall perceptions of patient safety	Procedures and systems are good at preventing errors and there is a lack of patient safety problems	4
9. Staffing	There are enough staff to handle the workload and work hours are appropriate to provide the best care for patients	4
10. Supervisor/manager expectations and actions promoting safety	Supervisors/managers consider staff suggestions for improving patient safety, praise staff for following patient safety procedures, and do not overlook patient safety problems	4
11. Teamwork across units	Hospital units cooperate and coordinate with one another to provide the best care for patients	4
12. Teamwork within units	Staff support one another, treat each other with respect, and work together as a team	4

The Hospital SOPS items use 5-point response scales of agreement (“Strongly disagree” to “Strongly agree”) or frequency (“Never” to “Always”). Each item’s percent positive score consists of the percentage of positive responses (“Agree” or “Strongly Agree”, “Most of the time” or “Always” to positively worded items) within a hospital. Prior to analyses, negatively worded items were reverse coded so that higher scores represent positive responses. Percent positive scores were calculated for composites by taking the average of the percent positive scores for the 3 or 4 items that make up the composite [20]. Rarely, hospitals exclude composite items from their survey or have such a small sample size that they do not receive a respective composite score. When an item is excluded, the composite score that includes that item is not calculated for that hospital.

The survey also includes a single-item measure that asks providers and staff to give their work area/unit a patient safety grade on a 5-point scale ranging from “Excellent” to “Failing”. The percent positive patient safety grade was calculated as the percentage of respondents who gave their work area/unit either a grade of “Excellent” or “Very good”. For these analyses we create a Hospital SOPS composite average index scores, which is the mean value of the 12 composite measures. All Hospital SOPS measures had the potential to range between 0 and 100% positive. This study utilized the Hospital SOPS 12 patient safety culture composites, the Hospital SOPS composite average score, and the overall patient safety rating.

#### Consumer Reports Hospital Safety Score

Consumer Reports (CR) is a U.S. based non-profit organization that provides its subscribers access to hospital ratings based on patient experience survey data and outcomes data provided by the Centers for Medicare and Medicaid Services (CMS) the Centers for Disease Control (CDC) and Prevention. Both CMS and CDC are federal agencies within the U.S. Department of Health and Human Services, which has the overall goal of protecting health of all Americans and providing essential health and human services. This data is used to reimburse and reward providers and health care settings that adhere to government policy. CR, using elements of this data, annually calculates a Hospital Safety Score based on five standardized component measures: 1) Hospital acquired infections, 2) Hospital-wide readmissions, 3) Communication about medications and discharge, 4) Appropriate use of scanning, and 5) Mortality. CR enlists the help of external expert reviewers for feedback on measure methodology and on how to turn raw federal data into hospital ratings. The technical details for how each measure is calculated can be found on the Consumer Reports Web site [21]. Higher scores indicate lower infection and readmissions rates, better communication about medications and discharge between staff and patients, more appropriate use of scanning, and lower mortality rates.

The CR Hospital Safety Score is the mean of these five equally weighted components. That mean is then linearly transformed to a scale of 0 to 100 (100 being the safest). CR chose these five measures to serve as indicators of a hospital’s commitment to patient safety and deliberately did not include process measures. Furthermore, the five components were selected because they represent a broad range of patient outcomes. When possible, federal data were statistically adjusted prior to public reporting to minimize differences among hospitals due to the types of patients they served.

#### Analysis dataset

The analysis dataset consisted of 164 hospitals out of the 419 potential hospitals that voluntarily submitted survey data to the AHRQ Hospital SOPS 2014 Comparative Database and were in the CR 2014 Hospital database. The 419 hospitals that existed in both databases were provided with a Data Use Agreement describing the proposed analyses and requesting permission to use their data. These analyses only include data from the 164 hospitals that authorized use of their data for this study. The Hospital SOPS data from these hospitals were collected from 2011 to 2013 and the CR data were collected between 2009 and 2013. All data were aggregated to the hospital level for analyses.

The 164 hospitals in the analytic dataset differed slightly in characteristics from the 6407 U.S. hospitals registered in the 2011 American Hospital Association (AHA) Annual Survey of Hospitals. Hospitals in the analytic dataset tended to be larger (i.e., more beds) compared with AHA-registered U.S. hospitals (Table 2). The majority of the hospitals in the analytic dataset were nonteaching (55%), though this was lower than the percentage of nonteaching AHA-registered U.S. hospitals (76%). Finally, the hospitals overrepresented nongovernment hospitals (93 versus 75% in the AHA dataset).

**Table 2:** Characteristics of 164 Hospitals in the Analysis Dataset vs. AHA National Dataset

	Analysis hospitals		2011 AHA-Registered U.S. hospitals	
Bed size	n	Percentage	n	Percentage
6–49 beds	6	4	2152	34
50–99 beds	30	18	1276	20
100–199 beds	41	25	1280	20
200–299 beds	34	21	684	11
300–399 beds	16	10	409	6
400–499 beds	16	10	201	3
500 or more beds	21	13	315	5
Total	164	100	6317	100

### Hospital characteristics as covariates

Hospital characteristics obtained from AHA data were examined as covariates or control variables: bed size (dummy coded as a categorical variable, with 49 beds or less as the comparison group), teaching status (coded as 1 for teaching and 0 for non-teaching), and ownership (coded as 1 for government and 0 for nongovernment). It is important to control for the impact of these variables because hospital characteristics have shown consistent associations with Hospital SOPS scores and also may be associated with the CR Safety Score [20].

### Analysis

Multivariate linear regressions were conducted to estimate the relationship between Hospital SOPS measures and the CR Hospital Safety Score, controlling for bed size, teaching status, and ownership. Regressions were conducted to estimate the relationships between Hospital SOPS measures and each of the five CR component measures: 1) Hospital acquired infections, 2) Hospital-wide readmissions, 3) Communication about medications and discharge, 4) Appropriate use of scanning, and 5) Mortality. Multivariate regression allows for the simultaneous analysis of multiple predictor variables (i.e., Hospital SOPS scores) and controls (bed size, teaching status, and ownership) on the dependent variable of interest (e.g., CR Safety Score). Using one Hospital SOPS measure per model avoids multicollinearity, which would occur if we included all Hospital SOPS measures in a single regression model. Multicollinearity can lead to biased standard errors and unreliable estimates of standardized regression coefficients. All analyses were completed using SAS 9.3.

## Results

### Descriptive statistics

The mean number of Hospital SOPS respondents per hospital was 856 (minimum, 34; maximum, 7,806). The mean Hospital SOPS response rate per hospital was 56% (minimum, 12%; maximum, 100%). As shown in Table 3, Hospital SOPS scores ranged from 43% positive response (Handoffs and transitions) to 81% positive response (Teamwork within units). The Hospital SOPS scores of the hospitals included in our analysis tended to be slightly lower than the full set of 653 hospitals in the 2014 Hospital SOPS Comparative Database (top of Table 3, last column).

**Table 3:** Descriptive Statistics for the 164 Hospitals in the Analysis Dataset

Hospital SOPS percent positive measures	Number	Mean (%)	SD (%)	Min (%)	Max (%)	Hospital SOPS 2014 database average (*n* = 653)
Hospital SOPS composite average score	163	63	5	49	82	64
1. Communication openness	164	62	5	48	75	62
2. Frequency of events reports	163	63	6	48	82	66
3. Feedback and communication about error	163	66	7	42	82	67
4. Handoffs and transitions	164	43	8	26	73	47
5. Management support for patient safety	164	69	8	43	90	72
6. Nonpunitive response to error	163	44	7	30	68	44
7. Organizational learning	164	72	6	54	89	73
8. Overall perceptions of patient safety	164	65	6	48	85	66
9. Staffing	163	55	7	38	80	55
10. Supervisor/manager expectations and actions promoting patient safety	164	75	4	66	87	76
11. Teamwork across units	163	57	8	36	84	61
12. Teamwork within units	163	81	4	70	90	81
Patient safety grade	163	75	8	57	92	76
Consumer Reports measures	Number	Mean	SD	Min	Max	Consumer Reports 2014 database average (*n* = 2,590)
CR Hospital Safety Score	164	54	8	30	75	51
1. Hospital acquired infections	164	3	1	1	6	3
2. Hospital-wide readmissions	164	3	1	1	5	3
3. Avoiding mortality	164	3	1	2	5	3
4. Communication about medications and discharge	164	2	1	1	4	2
5. Appropriate use of scanning	164	4	1	1	5	4

CR Hospital Safety Scores in the analysis dataset ranged from 29.86 to 74.98 with an average of 54.14, which was slightly higher than the overall CR database average of 50.91. Furthermore, all of the CR component measures in the analysis dataset, except the hospital acquired infections component measure, were higher than the CR database average (bottom of Table 3, last column).

### Covariates

Across the multiple regressions, the CR Hospital Safety Score and its five components were often negatively associated with teaching hospitals, such that teaching hospitals tended to have lower CR ratings. Ownership status and categorical bed size were generally not significantly related to the CR ratings.

### Relationships with CR Hospital Safety Score

Table 4 displays multivariate regression results. Higher Hospital SOPS composite average scores were associated with higher CR Hospital Safety Scores, controlling for bed size, ownership, and teaching status (β = 0.24, *p* < 0.05). Furthermore, nine of the 12 Hospitals SOPS composites were positively related with the CR Hospital Safety Score ranging from β = 0.17 to β = 0.29. The Hospital SOPS composite with the largest standardized coefficient was Teamwork within units, signifying that more positive staff perceptions of teamwork within units was associated with higher CR Hospital Safety Scores. The Hospital SOPS composites Teamwork across units and Overall perceptions of patient safety had the next largest relationships with CR Hospital Safety Scores (β = 0.23). The percentage of providers and staff giving their hospital a patient safety grade of “Excellent” or “Very good” was also related with higher CR Hospital Safety Scores (β = 0.22).

**Table 4:** Multiple Regression Standardized Coefficients with Hospital SOPS Measures predicting Consumer Report Hospital Safety Score

Hospital SOPS Percent Positive Scores	Consumer Reports					
	CR Hospital Safety Score	1) Hospital acquired infections	2) Hospital-wide readmissions	3) Avoiding Mortality	4) Communication about medications and discharge	5) Appropriate use of scanning
Hospital SOPS composite average score	0.24*	0.14	0.04	0.04	0.30*	0.09
1. Communication openness	0.22*	0.12	0.06	0.06	0.21*	0.10
2. Frequency of events reported	0.04	0.22*	−0.21*	0.03	0.09	−0.02
3. Feedback and communication about error	0.20*	0.24*	−0.05	0.08	0.18*	0.04
4. Handoffs and transitions	0.10	0.07	0.00	0.07	0.16	−0.03
5. Management support for patient safety	0.19*	0.19*	−0.02	−0.03	0.27*	0.06
6. Nonpunitive response to error	0.17*	−0.01	0.13	−0.10	0.27*	0.14
7. Organizational learning	0.14	0.14	−0.05	−0.05	0.25*	0.06
8. Overall perceptions of patient safety	0.23*	0.19*	0.02	0.06	0.31*	0.00
9. Staffing	0.19*	−0.03	0.10	0.08	0.29*	0.09
10. Supervisor/manager expectations and actions promoting patient safety	0.21*	0.12	0.08	−0.01	0.28*	0.06
11. Teamwork across units	0.23*	0.06	0.09	0.03	0.28*	0.12
12. Teamwork within units	0.29*	−0.03	0.17*	0.04	0.35*	0.22*
Patient safety grade	0.22*	0.09	0.02	0.07	0.31*	0.09

*n* = 164; * *p* < 0.05; standardized coefficients represent the strength of relationships between individual 2014 Consumer Reports measures and 2014 Hospital Survey on Patient Safety Culture percent positive measures at the hospital-level, controlling for bed size, ownership, and teaching status. Higher scores Consumer Reports scores indicate lower infection and readmissions rates, better communication about medications and discharge between staff and patients, more appropriate use of scanning, and lower mortality rates. Higher Hospital SOPS scores indicate better staff perceptions of patient safety culture

### Relationships with the five CR components

Higher hospital SOPS composite average scores were associated with better communication about medications and discharge (β = 0.30) (Table 4). For 10 of the 12 Hospital SOPS composites, higher patient safety culture scores were also associated with better communication about medications and discharge component (range β = 0.18 to 0.35); the strongest relationship was with Teamwork within units. Higher patient safety culture scores for four composites were also associated with lower hospital acquired infections, ranging from β = 0.19 to β = 0.24.

Two Hospital SOPS composites were related to the CR component Hospital-wide readmissions: 1) More frequent reporting of patient safety events was associated with higher readmission rates (β = −0.21), which was in a direction that was opposite of our hypothesized relationship, and 2) better Teamwork within units was associated with more appropriate use of scanning (β = 0.17). None of the Hospital SOPS measures were related to the CR component Mortality.

### Relationships with CR Communication about medications and discharge subcomponents

Of the five CR components, the patient experience component on Communication about medications and discharge had the strongest and most consistent relationships with Hospital SOPS measures. Therefore, we conducted additional analysis to better understand what was driving these relationships.

The CR component Communication about medications and discharge is made up of two patient experience subcomponents: 1) Communication about medications and 2) Communication about discharge; each subcomponent is derived from two CAHPS survey items. The CR subcomponent Communication about medications was derived from the following CAHPS items: 1) Before giving you any new medicine, how often did hospital staff tell you what the medicine was for? 2) Before giving you any new medicine, how often did hospital staff describe possible side effects in a way you could understand?

The CR Communication about discharge was derived from the Hospital CAHPS items: 1) During this hospital stay, did doctors, nurses or other hospital staff talk with you about whether you would have the help you needed when you left the hospital? 2) During this hospital stay, did you get information in writing about what symptoms or health problems to look out for after you left the hospital? Information on how these subcomponents were calculated can be found on the Consumer Reports Web site [21].

We conducted post hoc linear regression analysis to determine which, if either, of the two communication subcomponents 1) Communication about medications and 2) Communication about discharge, was driving the relationship with the Hospital SOPS measures, even when controlling for bed size, ownership, and teaching status (Table 5). All 12 Hospital SOPS composite scores (range: β = 0.24 to β = 0.44) and the Hospital SOPS patient safety grade (β = 0.44) were positively related to the CR subcomponent Communication about medications indicating that higher staff perceptions of patient safety culture were related to higher patient perceptions of how well hospital staff communicate about medication. Five Hospital SOPS composites were positively related to the CR subcomponent Communication about discharge, ranging from β = 0.17 to β = 0.24.

**Table 5:** Multiple Regression Standardized Coefficients with Hospital SOPS Measures Predicting Subcomponents of the Consumer Report Hospital Safety Score

	Consumer Reports Communication subcomponents	
Hospital SOPS Percent Positive Measures	Communication about discharge	Communication about medications
1. Communication openness	0.11	0.28*
2. Frequency of events reported	−0.06	0.21*
3. Feedback and communication about error	0.05	0.27*
4. Handoffs and transitions	0.04	0.24*
5. Management support for patient safety	0.13	0.35*
6. Nonpunitive response to error	0.16	0.32*
7. Organizational learning	0.18*	0.26*
8. Overall perceptions of patient safety	0.20*	0.36*
9. Staffing	0.17*	0.35*
10. Supervisor/manager expectations and actions promoting patient safety	0.21*	0.29*
11. Teamwork across units	0.14	0.36*
12. Teamwork within units	0.24*	0.39*
Hospital SOPS composite average score	0.15	0.38*
Patient safety grade	0.12	0.44*

*n* = 164; * *p* < 0.05; standardized coefficients represent the strength of relationships between individual 2014 Consumer Reports measures and 2014 Hospital Survey on Patient Safety Culture percent positive measures at the hospital-level, controlling for bed size, ownership, and teaching status

## Discussion

This is the first study that has explored the relationship between hospital providers’ and staff perceptions of patient safety culture and the publicly reported, consumer-oriented CR Hospital Safety Score, which was derived from publicly available hospital data. We found that hospitals where providers and staff have more positive perceptions of patient safety culture tended to have higher CR Hospital Safety Scores. Our results also show that higher frequency of events reported is related to higher hospital-wide readmission rates. For the most part, these findings suggest that both the Hospital SOPS and the CR Hospital Safety Score are valid measures that generally complement each other. Given that Medicare-certified hospitals are reimbursed for providing high quality services and, in particular, lower readmission rates, future research might investigate if improving one’s patient safety culture might also lead to improvements in aspects of care that would lead to higher future CMS reimbursement rates.

Drilling down further to help determine what drives these findings, the better communication about medications with patients was most often related to higher Hospital SOPS measures. These findings are consistent with Sorra et al. [17], which found significant relationships between 10 of the 12 Hospital SOPS composites and the Hospital CAHPS Communication about medicine item while only one Hospital SOPS composite, Frequency of events reported, was related to the Communication about discharge item. In addition, more positive Teamwork within units was related to lower hospital-wide readmissions, better communication about medications and discharge, and more appropriate use of scanning.

While not the primary focus of this research, it is interesting to note that teaching hospitals tended to have lower CR Hospital Safety Scores. These findings are consistent with previous studies that found AHRQ patient safety indicator rates, which distinguish adverse events resulting from medical intervention, are higher among teaching hospitals [14, 22]. In addition, teaching hospitals have historically reported lower patient safety culture scores [23].

There are several limitations to this analysis. We were able to conduct analysis on only 164 hospitals that voluntarily agreed to participate in the study and also independently administer the Hospital SOPS survey. These hospitals may be more actively addressing patient safety and quality issues compared with hospitals that declined participation or do not administer Hospital SOPS. However, this is less likely given the broad range of Hospital SOPS composite scores. Larger, non-government hospitals are overrepresented compared to non-participating hospitals in this analysis, so findings may not be generalizable to the entire U.S. hospital population.

In addition, hospitals do not provide staff position census numbers when submitting survey data to the Comparative Database and collect survey data using either paper, online, or both modes. It is also unknown if hospitals equally targeted or incentivized staff to complete surveys. Therefore, submitted staff responses may not be representative of their respective hospitals.

A third limitation is that this study was cross-sectional, and we cannot directly measure any causal relationship between patient safety culture and the outcomes aggregated in the CR Safety Score. Since we hypothesized that patient safety culture would be related to hospital outcomes, ideally patient safety culture results would be collected preceding or concurrently with hospital outcomes data. However, a portion of the outcomes data used to calculate CR Safety Scores was collected up to two years earlier than the Hospital SOPS data; furthermore, a portion of the mortality component was collected up to four years prior to Hospital SOPS administration. This measurement “noise” could have affected our findings or lack thereof. However, the Hospital SOPS timeframe does coincide with the CR component measures that had the strongest relationships (i.e., Communication about medications and discharge and Hospital acquired infections).

Future analysis should more closely examine how nationally standardized outcome measures, as well as other CR ratings, relate to Hospital SOPS measures. In addition, research examining the relationships between patient safety ratings from other companies (e.g., Leapfrog, U.S News and World Report, and Healthgrades) and provider and staff perceptions of patient safety culture might help patients and hospital leaders further understand how these different measures of patient safety are related patient safety and quality [24]. Additional research on the relationships between medical students, patient safety culture, and preventable adverse events would be prudent.

## Conclusions

In conclusion, we have discovered relationships between AHRQ Hospital SOPS measures and the CR Safety Score and its components. We found statistically significant relationships showing that higher Hospital SOPS composite scores are associated with higher CR Hospital Safety Scores. Furthermore, our analysis of the CR Hospital Safety Score components suggests that the main driver of these relationships was the patient experience communication component collected by the CAHPS Hospital survey. This analysis may lay the groundwork for future research examining the relationships between hospital provider and staff perceptions of patient safety culture and publicly reported patient safety scores.

## Abbreviations

AHA: American Hospital Association
AHRQ: The Agency for Healthcare Research and Quality
CAHPS: Consumer Assessment of Healthcare Providers and Systems (CAHPS®)
CDC: The Centers for Disease Control
CMS: The Centers for Medicare and Medicaid
CR: Consumer Reports
SOPS: Surveys on Patient Safety Culture (SOPS^TM^)
